# Trans-limb embolization for treatment of Type 2 endoleak post EVAR: Case report

**DOI:** 10.1016/j.ijscr.2021.106238

**Published:** 2021-07-26

**Authors:** E. Dinoto, F. Ferlito, F. Urso, S. Evola, G. Bajardi, F. Pecoraro

**Affiliations:** a Vascular Surgery Unit - AOUP Policlinico ‘P. Giaccone’, Palermo, Italy; b Department of Surgical, Oncological and Oral Sciences, University of Palermo, Italy; c Unit of Cardiology, Department of Health Promotion, Mother and Child Care, Internal Medicine and Medical Specialties (ProMISE) 'G. D'Alessandro', University Hospital Paolo Giaccone, University of Palermo, Palermo, Italy

**Keywords:** Endoleak type 2, Coil embolization, Aneurysm, Case report

## Abstract

**Introduction:**

Type 2 endoleaks (T2EL) occur after 10%–25% of endovascular abdominal aortic aneurysm repairs and increase the risk factor of endograft repair failure and rupture. Herein we report a case of endovascular treatment of T2EL where we performed a trans-limb embolization.

**Presentation of case:**

A 63-years-old male previously treated for AAA with endovascular aortic aneurysms repair (EVAR), showed an angio-CT scan followup with a type 2 endoleak fed from inferior mesenteric artery (AMI) with growth of AAA greater of 1 cm than preoperative CT-scan and increase of chronic lumbar pain. Due to high risk of rupture was performed a trans-limb embolization with complete sealing. The 6 months CT-angiography showed complete type 2 endoleak exclusion without changes of AAA.

**Discussion:**

The risk of aneurysm rupture in the presence of an isolated T2EL is exceptionally low. However, when a persistent T2EL is associated with a significant sac size increase, commonly considered as at least 5 mm over 6 months, should be treated. Detachable coils are repositionable, allowing an extremely precise deployment and subsequent embolization of different targets.

**Conclusion:**

In this experience trans-limb embolization was feasible and this tool should be taken in account especially when no other surgical options exists.

## Highlights

• Type 2 endoleaks (T2EL) are a risk factor for endograft repair failure and rupture.
• T2EL are the most common endoleaks and remain the main cause of repeat intervention.
• Trans-limb embolization is an innovative technique for T2EL without standard access.
• Concerto are detachable coils repositionable allowing an extreme precise deployment.

## 1 Introduction

Type 2 endoleaks (T2EL) occur after 10%–25% of endovascular abdominal aortic aneurysm repairs and are a risk factor for endograft repair failure and rupture [1]. These are complications given by inversion of blood flow from collateral arteries [2]. Approximately 80% e 90% of T2ELs undergo spontaneous resolution [3]. The remaining portion can increase the diameters of aneurysm sac with risk of rupture and consequent hemorrhage [4].

Herein we report a case of endovascular treatment of T2EL where we performed a trans-limb embolization.

This work has been written in accordance with the SCARE criteria [5].

## 2 Case report

A 63-years-old male with hypertension, diabetes mellitus was referred for incidental diagnosis of Abdominal Aortic Aneurysm (AAA) after Ultrasoundoppler (US), performed for abdominal pain. The angio-CT scan confirmed the presence of AAA with maximum diameter of 6.5 cm. At admission, his physical examination revealed regular heart rate of 80 beats/min, blood pressure of 140/70 mm Hg and temperature of 36.8 °C. At history, he referred a story of lumbar pain by herniated disk.

**Fig. 1:**
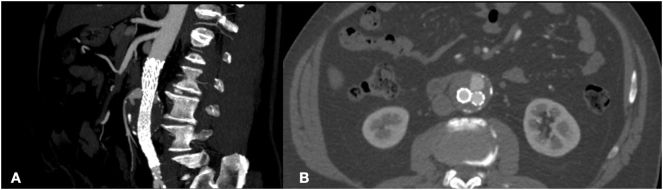
CT-angiography at 2 months after EVAR showing type 2 endoleak fed from inferior mesenteric artery in sagittal plane (A) and traversal plane (B).

**Fig. 2:**
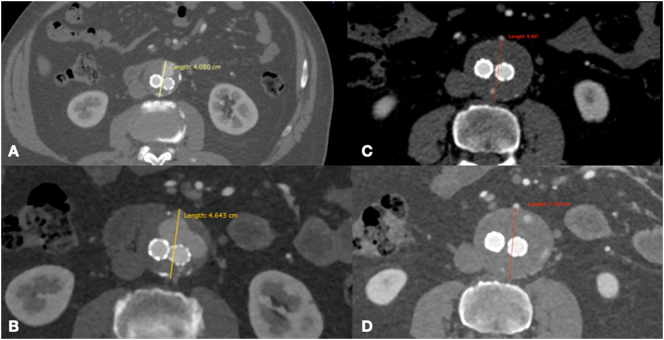
CT-angiography at 6 months showing a worsening of type 2 endoleak (A and B) with growth of AAA greater of 0.5 cm (C and D).

The patient was considered fit for conventional endovascular approach and under local anesthesia was performed an endovascular aortic aneurysms repair (EVAR) with release of Gore Endoprosthesis 28 mm (W.L. Gore and Associates, Inc., Flagstaff, Ariz) without intraoperative visualization of endoleak at final angiography check. The patient was discharged on the 3rd postoperative day. The 2 months CT-angiography showed a type 2 endoleak fed from inferior mesenteric artery (AMI) without changes of diameters in Aneurysm Sac (Fig. 1). At 6 months followup, CT-Angiography showed a worsening of T2EL with growth of AAA greater of 0.5 cm and increase of chronic lumbar pain (Fig. 2). Thus, in light of these findings we chose to perform a second Endovascular procedure.

**Fig. 3:**
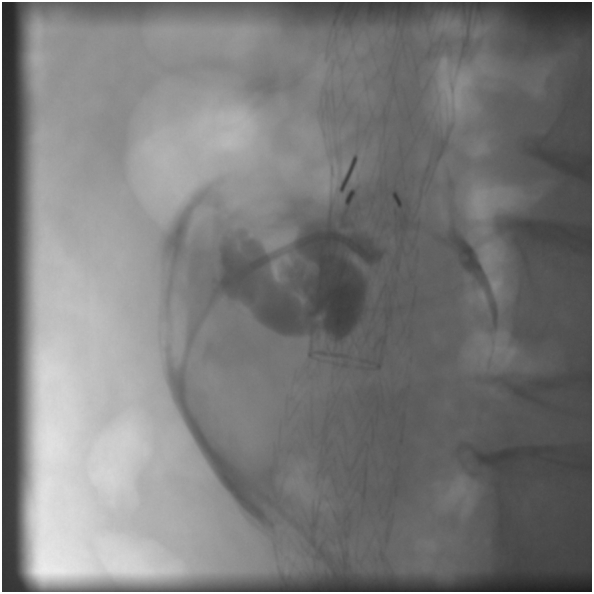
Intraoperative Endoleakogram.

**Fig. 4:**
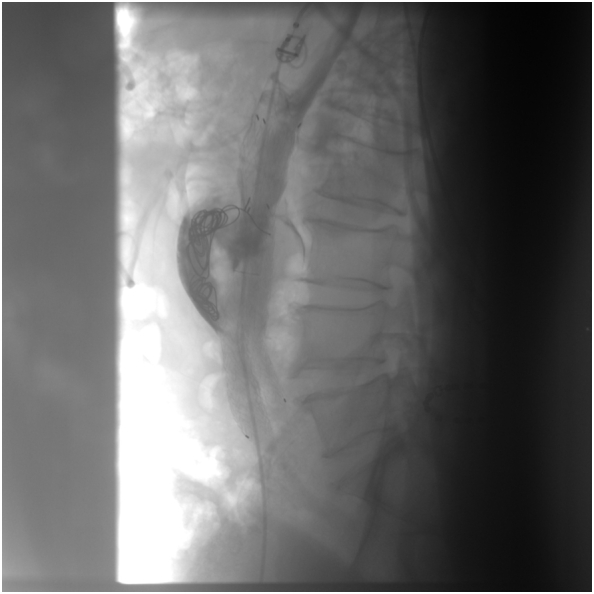
Intraoperative Endoleakogram after release of Concerto Helix Detachable Coil System.

Under local anesthesia, a right femoral access was gained and a 5Fx25cm sheath placed. The sheath tip was placed a few centimeters below the end of the iliac endograft. The 0.014” Command wire (Abbott Vascular, Park, Illinois) was passed between iliac Endoprosthesis right leg and arterial wall due to enter aneurysm sac. To allow the transition of 5F Ber catheter (Cordis, Miami, FL), we performed a pre-dilatation with Amphirion deep balloon 2 × 40 mm (Medtronic, Inc., Minneapolis, MN, US). Endoleakogram by 5 Fr Ber confirmed T2EL and a 0.018 microcatheter (Rebar18; Medtronic, Inc., Minneapolis, MN, US) was advanced coaxially under fluoroscopic visualization near to origins of IMA (Fig. 3). Subsequently through the 0.018 microcatheter, two Concerto Helix Detachable Coil System (16 mm × 40 cm - the first and 14 mm × 30 cm - the second) (Medtronic, Inc., Minneapolis, MN, US) were released inside the aneurysm sac to fill the type 2 endoleak. The control angiography confirmed the type 2 endoleak sealing after coil embolization (Fig. 4). At 6 h from the intervention the lumbar pain was reduced; the patient was discharged on the 3rd postoperative day.

**Fig. 5:**
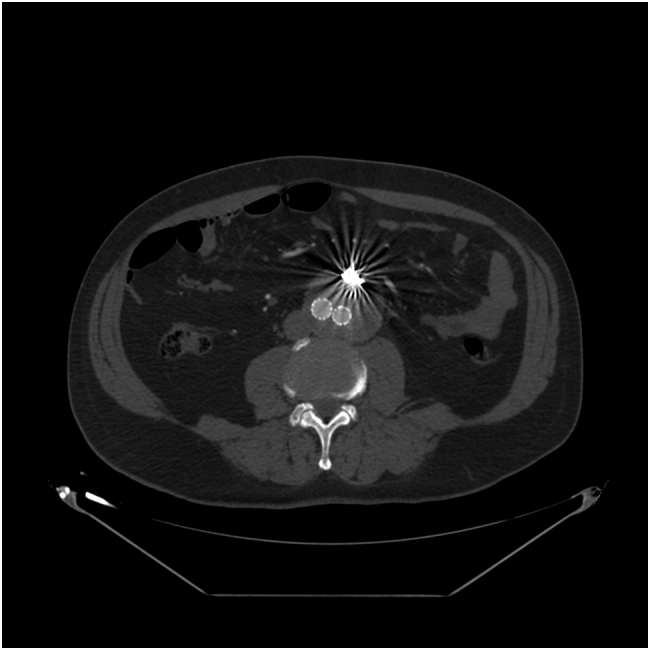
CT-angiography at 6 months after embolization showing complete type 2 endoleak sealing.

The 6 months CT-angiography, after last procedure, showed complete type 2 endoleak sealing without changes of AAA (Fig. 5). The 12 months Colordoppler Ultrasound showed a lack of growth in AAA.

## 3 Discussion

Endoleaks are EVAR-related complications occurring immediately or during the follow-up [6]. T2EL are the most common endoleaks following EVAR and remain the main cause of repeat intervention [7]. They are caused by retrograde blood flow into the sac from branches of the endograft covered native aorta or iliac vessels. There is usually one dominant inflow artery, most commonly the IMA or a lumbar artery, and often one or more outflow arteries [8]. T2EL are inherently low flow and are often transient, resolving following thrombosis of the aneurysm sac and reversal of anticoagulation. The risk of aneurysm rupture in the presence of an isolated T2EL is exceptionally low [9]. The current consensus is that one should treat a persistent T2EL when they are associated with a significant sac size increase, commonly considered as at least 5 mm over 6 months [10], [11]. In the absence of an enlarging sac size, for asymptomatic patients observation could be an option; for symptomatic or ruptured cases alternative therapeutic options has to be considered. Embolization is the main treatment for T2EL. The aim of intervention is to obliterate the endoleak cavity. There are a variety of embolization techniques depending on the anatomy of the artery supplying the endoleak and the available route to access the endoleaks.

The most common technique is transarterial catheterization of the dominant feeding vessel via communicating arteries supplying the vessel. This approach is performed under conscious sedation and requires an accessible route from an aortoiliac vessel, via collaterals to the vessel feeding the endoleak and ideally the endoleak cavity itself. The technical success of this approach is limited if the responsible feeding vessel cannot be cannulated or if a viable path to the endoleak cavity cannot be found [12]. Alternative is the direct percutaneous puncture of the aneurysm sac. There are only two papers that have specifically reported the outcomes of direct sac puncture embolization. In the larger of these studies, Zener et al. (2018) reported on 33 transabdominal embolizations in 30 patients using a range of embolic agents with a technical success rate of 97% and clinical success of 85%, defined as freedom from sac growth [13], [14], [15]. Other technique is the transcaval access into the endoleak cavity that is achieved by using an angled-tip catheter and an angled sheathed needle (e.g. TIPSS set) to penetrate the Inferior Vena Cava wall and enter the endoleak cavity. The largest cohort included 29 patients, reported by Giles et al., with technical success achieved in 90% and no significant adverse events, although 5 patients required reintervention [16]. In this case reported we chose an innovative approach, represented by trans-limb embolization, an innovative technique useful, especially, when aneurysm sac cannot be accessed by the standard transarterial route. Once access into the paraendograft space is obtained, the catheter and wire are advanced superiorly using standard catheter–guidewire manipulation techniques between the graft and the artery wall until access to the sac thrombus is achieved. After performing an endoleakogram to define the anatomy of the endoleak, any visible and accessible feeding vessels are embolised with a liquid embolic or as in our experience with coils. Using this technique, Coppi and colleagues reported successful embolization of the sac in 16 of 17 patients using a 9F sheath, with one adverse event of a procedural type Ib endoleak [17], [18]. In practice, procedural success is limited by difficulty in accessing the paraendograft space and accessing the endoleak nidus even when the sac thrombus has been accessed. Embolization of the sac thrombus if the nidus cannot be accessed is of no benefit [8], [19], [20]. In this regard, it was beneficial in trans-limb embolization, the use of Concerto Helix Detachable Coil System (Medtronic, Inc., Minneapolis, MN, US). On the other hand, detachable coils are repositionable, allowing an extremely precise deployment and subsequent embolization of different targets [21].

In this single case experience the 6-months CT angiography and Colordoppler Ultrasound after 12-months showed complete AAA exclusion.

In patients with T2EL followup is important. Our protocol includes after EVAR a CT angiography at 2-months with a new CT-scan if a T2EL is found during the first check. After embolization or spontaneous resolution followup consist of a CT angiography at 6-months and Colordoppler Ultrasound check every six months. In cases of doubt Colordoppler Ultrasound may be complemented by contrast-enhanced ultrasound or new CT angiography [4], [19], [22].

## 4 Conclusion

T2EL is a complication post EVAR where the followup is mandatory. Minimally invasive procedures are useful and safe to treat these lesions. In literature there are few papers reporting description of the technique, with analysis of outcomes about trans-limb embolization and more experience is necessary. However, in this report trans-limb embolization was feasible and its advantages were mainly its low invasiveness, ease of use and the possibility to have an extremely precise deployment due to the use of repositionable coils. This technique should be taken in account especially when no other surgical options exists.

## Funding

None.

## Ethical approval

None.

## Consent

Written informed consent was obtained from the patient for publication of this case report and accompanying images. A copy of the written consent is available for review by the Editor-in-Chief of this journal on request.

## Author contribution

Ettore Dinoto: study concept, design, data collection, data analysis, interpretation, writing the paper, final approval of the version to be submitted, guarantor.

Felice Pecoraro: study concept, design, data collection, data analysis, interpretation, writing the paper, final approval of the version to be submitted.

Francesca Ferlito: study concept, design, data collection, data analysis, interpretation, final approval of the version to be submitted.

Francesca Urso: study concept, design, data collection, final approval of the version to be submitted.

Salvatore Evola: study concept, design, data collection, final approval of the version to be submitted.

Guido Bajardi: study concept, design, data collection, data analysis, interpretation, final approval of the version to be submitted.

## Guarantor

Ettore Dinoto.

## Provenance and peer review

Not commissioned, externally peer-reviewed.

## Declaration of competing interest

The authors have no ethical conflicts to disclose.

## References

[1] Hicks R.M., Vartanian S.M., Lehrman E.D. (2021 May 24). Transvenous type 2 endoleak embolization using intravascular ultrasound guidance via a left-sided inferior vena cava. J. Vasc. Interv. Radiol..

[2] Pecoraro F., Pakeliani D., Dinoto E., Bajardi G. (2017 Apr). Endovascular treatment of large and wide aortic neck: case report and literature review. Gen. Thorac. Cardiovasc. Surg..

[3] Sheehan M.K., Ouriel K., Greenberg R., McCann R., Murphy M., Fillinger M., Wyers M., Carpenter J., Fairman R., Makaroun M.S. (2006 Apr). Are type II endoleaks after endovascular aneurysm repair endograft dependent?. J. Vasc. Surg..

[4] Pecoraro F., Bracale U.M., Farina A., Badalamenti G., Ferlito F., Lachat M., Dinoto E., Asti V., Bajardi G. (2019 Apr). Single-center experience and preliminary results of intravascular ultrasound in endovascular aneurysm repair. Ann. Vasc. Surg..

[5] Agha R.A., Franchi T., Sohrabi C., Mathew G., for the SCARE Group (2020). The SCARE 2020 guideline: updating consensus Surgical CAse REport (SCARE) guidelines. Int. J. Surg..

[6] Dinoto E., Ferlito F., Mirabella D., Tortomasi G., Bajardi G., Pecoraro F. (2021 May 26). Type 1A endoleak detachable coil embolization after endovascular aneurysm sealing: case report. Int. J. Surg. Case Rep..

[7] Yu H., Isaacson A.J., Dixon R.G. (2017). Comparison of type II endoleak embolizations: embolization of endoleak nidus only versus embolization of endoleak nidus and branch vessels. J. Vasc. Interv. Radiol..

[8] Ameli-Renani S., Pavlidis V., Morgan R.A. (2020 Dec). Secondary endoleak management following TEVAR and EVAR. Cardiovasc. Interv. Radiol..

[9] Sidloff D.A., Gokani V., Stather P.W. (2014). Type II endoleak: conservative management is a safe strategy. Eur. J. Vasc. Endovasc. Surg..

[10] Chung R., Morgan R.A. (2015). Type 2 endoleaks post-evar: current evidence for rupture risk, intervention and outcomes of treatment. Cardiovasc. Interv. Radiol..

[11] Pecoraro F., Dinoto E., Mirabella D., Ferlito F., Farina A., Pakeliani D., Lachat M., Urso F., Bajardi G. (2020 Dec). Endovascular treatment of spontaneous and isolated infrarenal acute aortic syndrome with unibody aortic stent-grafts. World J. Surg..

[12] Chung R., Morgan R. (2014). Technical note: “remote” transarterial embolization technique of lumbar artery type 2 endoleaks with onyx. EJVES Extra.

[13] Zener R., Oreopoulos G., Beecroft R. (2018). Transabdominal direct sac puncture embolization of type II endoleaks after endovascular abdominal aortic aneurysm repair. J. Vasc. Interv. Radiol..

[14] Pecoraro F., Corte G., Dinoto E., Badalamenti G., Bruno S., Bajardi G. (2016 Sep-Oct). Clinical outcomes of Endurant II stent-graft for infrarenal aortic aneurysm repair: comparison of on-label versus off-label use. Diagn. Interv. Radiol..

[15] Carrafiello G., Ierardi A.M., Radaelli A. (2016). Unenhanced cone beam computed tomography and fusion imaging in direct percutaneous sac injection for treatment of type II endoleak: technical note. Cardiovasc. Interv. Radiol..

[16] Giles K.A., Fillinger M.F., De Martino R.R. (2015). Results of transcaval embolization for sac expansion from type II endoleaks after endovascular aneurysm repair. J. Vasc. Surg..

[17] Ameli-Renani S., Pavlidis V., Mailli L. (2016). Transiliac paraendograft embolization of type 2 endoleak: an alternative approach for endoleak management. Cardiovasc. Interv. Radiol..

[18] Coppi G., Saitta G., Coppi G., Gennai S., Lauricella A., Silingardi R. (2014 Apr). Transealing: a novel and simple technique for embolization of type 2 endoleaks through direct sac access from the distal stent-graft landing zone. Eur. J. Vasc. Endovasc. Surg..

[19] Bracale U.M., Corte G., Del Guercio L., Pecoraro F., Dinoto E., La Rosa G., Porcellini M., Bracale G., Bajardi G. (2012 Nov-Dec). Endovascular treatment of abdominal aortic anastomotic pseudoaneurysm. the experience of two centers. Ann. Ital. Chir..

[20] Ierardi A.M., Franchin M., Fontana F. (2018). The role of ethylene– vinyl alcohol copolymer in association with other embolic agents for the percutaneous and endovascular treatment of type ia endoleak. Radiol Med..

[21] Bracale U.M., Narese D., Ficarelli I., Laurentis M., Spalla F., Dinoto E., Vitale G., Solari D., Bajardi G., Pecoraro F. (2017 Jan-Feb). Stent-assisted detachable coil embolization of wide-necked renal artery aneurysms. Diagn. Interv. Radiol..

[22] Dinoto E., Pecoraro F., Farina A., Viscardi A., Bajardi G. (2020). Simultaneous endovascular treatment of synchronous symptomatic acute type B aortic dissection and large infrarenal aortic aneurysm. Technical tips and case report. Int. J. Surg. Case Rep..

